# Evaluation of Commercial Immunoassays for Rubella Virus IgG Detection in Low-Antibody Sera Using a Recombinant Immunoblot as a Reference Method

**DOI:** 10.3390/microorganisms14010058

**Published:** 2025-12-26

**Authors:** Carmen Ortega, Antonio Sampedro-Padilla, Pablo Mazuelas, Jose Serrano, Ana Abreu, Juan Antonio Reguera, Javier Rodríguez-Granger, Fernando Cobo, Juan Francisco Gutiérrez-Bautista, Antonio Sampedro

**Affiliations:** 1 Servicio de Microbiología, Virgen de las Nieves University Hospital, 18014 Granada, Spain; mariac.ortega.gavilan.sspa@juntadeandalucia.es (C.O.); josem.serrano.romero.sspa@juntadeandalucia.es (J.S.); anab.abreu.sspa@juntadeandalucia.es (A.A.); jantonio.reguera.sspa@juntadeandalucia.es (J.A.R.); javierm.rodriguez.sspa@juntadeandalucia.es (J.R.-G.); antonioj.sampedro.sspa@juntadeandalucia.es (A.S.); 2 Zaidín Sur Health Center, Andalusian Health Service, Granada Metropolitan District, 18007 Granada, Spain; ajesus.sampedro.sspa@juntadeandalucia.es; 3 Servicio de Microbiología, Virgen de la Victoria University Hospital, 29010 Malaga, Spain; josep.mazuelas.sspa@juntadeandalucia.es; 4 Instituto de Investigación Biosanitaria de Granada (ibs.GRANADA), 18012 Granada, Spain; 5 Servicio de Análisis Clínicos e Inmunología, University Hospital Virgen de las Nieves, 18014 Granada, Spain; 6 Departamento de Bioquímica, Biología Molecular e Inmunología III, University of Granada, 18016 Granada, Spain

**Keywords:** rubella IgG, immunoassay variability, low-titer vaccinated populations, serological assay harmonization, rubella immunity assessment

## Abstract

Rubella virus (RV) IgG quantification is essential for verifying immunity, particularly in prenatal care. However, substantial variability exists among commercial immunoassays, especially when testing low-antibody sera. In this study, we evaluated five commercial assays—four chemiluminescent immunoassays (CLIAs) and one Enzyme-linked Immunosorbent Assay (ELISA)—using a recombinant immunoblot (IB) as the reference method. A panel of 137 serum samples with low or undetectable IgG levels was analyzed. Sensitivity ranged from 19.6% to 70.1%, while specificity exceeded 94%. Only 18.6% of immunoblot-positive samples tested positive across all assays. Marked quantitative differences were observed, with the Atellica assay yielding the highest titers and Alinity the lowest. Reclassifying equivocal results as positive improved concordance without compromising specificity. These findings suggest that current cut-off values, derived from post-infection sera, may be inadequate for vaccinated populations. A single universal threshold may lead to misclassification and underestimation of immunity. Harmonization of assay calibrations, antigenic targets, and interpretation criteria is urgently needed to ensure reliable rubella immunity assessments in clinical and public health settings.

## 1. Introduction

The rubella virus (RV) is a Togavirus belonging to the Rubivirus genus, causing an exanthematous disease in susceptible individuals. Rubella infection is often subclinical or presents with mild symptoms such as fever, nausea, and a transient rash; however, complications can occur, particularly in adult women. The most common complication involves arthritis affecting the fingers, wrists, and knees, typically lasting about one month. Occasionally, more severe sequelae may arise, including encephalitis and other neurological manifestations. Nevertheless, the most significant concern associated with rubella infection is its teratogenic potential in congenital infections. Effects on the fetus range from spontaneous abortion or fetal death to congenital rubella syndrome (CRS) [1].

Vaccination is the most effective measure for rubella elimination. The seroconversion rate after two doses of the measles–mumps–rubella (MMR) vaccine approaches 99%, and antibodies persist for at least 21 years [2]. Vaccination coverage of at least 95% of the population is required to interrupt transmission [3]. Currently, rubella and CRS are rare or have been eliminated in countries with effective immunization programs; in 2020, the WHO verified elimination in 93 countries [4].

Although the immune response to vaccination mimics wild-type infection, antibody levels are generally lower and wane more rapidly than those elicited by natural infection [5]. Consequently, in populations vaccinated decades ago, lower RV IgG levels are observed among younger cohorts [6,7]. To maintain adequate immunity rates, assessing rubella immunity during the preconception period or in pregnant women without vaccination records remains essential, enabling postpartum immunization if necessary [8].

Determining protective immunity to rubella in the post-vaccination era has become increasingly complex. The success of the rubella vaccine lies in its ability to elicit both humoral and cellular immune responses [9]. However, assessing cellular immunity is laborious and limited to specialized laboratories, whereas measuring circulating antibodies is relatively simple. Therefore, rubella immunity is routinely evaluated through the quantification of RV IgG antibodies using commercial immunoassays (CIAs). These assays are calibrated against the WHO International Standard (RUBI-1-94) and express results in International Units per millilitre (IU/mL), with a conventional cut-off of 10 IU/mL defining seropositivity [10].

However, RUBI-1-94 was derived from sera obtained after natural infection, not vaccination, which may limit its traceability in vaccinated populations [11]. Moreover, the calibration processes, antigenic composition, and analytical platforms differ among manufacturers, leading to a lack of harmonization across assays [12,13]. Comparative studies have shown that discrepancies in antibody quantification are especially frequent in samples with low IgG concentrations—often near the diagnostic cut-off [12,13]. Such inconsistencies can complicate clinical interpretation, particularly in prenatal screening, and raise questions about the adequacy of a universal cut-off value for all assays.

The objective of this study was to compare the performance and concordance of five commercial CIAs for RV IgG detection using a recombinant immunoblot as the reference method. Our findings aim to contribute to ongoing efforts to harmonize rubella serological assays and to refine the interpretation of IgG levels, especially in samples with low antibody concentrations.

However, few studies have specifically examined the performance of widely used commercial immunoassays in low-titer sera from vaccinated populations, where discrepancies are most likely to occur. Addressing this gap is essential for improving the reliability of rubella immunity assessment in both clinical and public health settings.

## 2. Materials and Methods

### 2.1. Samples

A total of 137 serum samples were included in this study. The samples were collected between May and September 2025 from routine diagnostic testing at the Microbiology Departments of Virgen de las Nieves University Hospital (Granada, n = 90) and Virgen de la Victoria University Hospital (Málaga, n = 47), and were anonymized prior to analysis. Inclusion criteria were the availability of sufficient sample volume and RV IgG concentrations ≤20 IU/mL in preliminary testing. The initial assays used at each hospital were Alinity i Rubella IgG (Abbott Diagnostics) at Virgen de las Nieves and Atellica IM Rubella IgG (Siemens Healthcare) at Virgen de la Victoria. The samples came from pregnant women (n = 76), children (n = 15), and healthcare workers (n = 46) who were routinely tested for rubella immunity. Vaccination history was unknown for all participants. This study was designed as a method-comparison analysis rather than a seroprevalence survey. Therefore, the sample set was not intended to be a random representation of the general population. Instead, we deliberately enriched the panel with sera showing negative or low-positive results close to the decision limit in routine screening (≤20 IU/mL), where inter-assay discrepancies are most clinically relevant.

After the initial testing, the samples were stored at −20 °C. Before testing, frozen samples were thawed and clarified by centrifugation at 2000× *g* for 10 min. Thawed samples were stored at 4 °C for up to 5 days. All comparative testing was performed within this refrigerated window to minimize pre-analytical variability in low-reactivity samples.

All procedures were conducted in accordance with the Declaration of Helsinki.

### 2.2. Commercial Assays

Five CIAs were evaluated for the quantitative detection of anti-RV IgG antibodies. Four were chemiluminescent immunoassays (CLIAs)—Alinity i Rubella IgG (Abbott Diagnostics, Chicago, IL, USA), Atellica IM Rubella IgG (Siemens Healthcare, Munich, Germany), Liaison Rubella IgG (DiaSorin, Torino, Italy), and Rubella VIRCLIA IgG (Vircell, Granada, Spain)—and one was an Enzyme-linked Immunosorbent Assay (ELISA), Anti-Rubella Virus IgG ELISA (Euroimmun, Lübeck, Germany). The five assays were selected a priori based on their availability in the participating laboratories and their use as automated platforms for rubella IgG screening in routine practice. By including four CLIA-based systems from different manufacturers and one ELISA, we aimed to capture inter-assay variability that may affect the interpretation of low-titer samples across commonly used diagnostic platforms.

All assays were performed according to the manufacturers’ instructions on their respective automated platforms. The immunoassays evaluated use proprietary rubella virus antigen preparations and manufacturer-specific calibration schemes. Differences in assay architecture, calibration, and the definition of the equivocal zone can contribute to quantitative changes and qualitative discrepancies, especially in low-titer samples.

The key analytical and interpretative characteristics of each assay (platform, method type, and negative/equivocal/positive breakpoints) are summarized in Supplementary Table S1.

Results were expressed in international units per millilitre (IU/mL) and calibrated against the WHO International Standard RUBI-1-94. According to manufacturer-defined criteria, CLIA results ≤4.9 IU/mL were considered negative, 5.0–9.9 IU/mL equivocal, and ≥10.0 IU/mL positive. For the Euroimmun ELISA, values ≤ 7.9 IU/mL were considered negative, 8.0–10.9 IU/mL equivocal, and ≥11 IU/mL positive. Internal quality controls provided by the manufacturers were included in every analytical run. Discrepant or equivocal samples were retested in duplicate to confirm reproducibility.

### 2.3. Reference Immunoblot Assay

This immunoblot (IB) assay has recently been used as a reference method in studies assessing the sensitivity of RV IgG assays [12]. The IB enables the detection of antibodies against specific RV proteins: envelope proteins E1 and E2, capsid protein C, and the E1–E2 antigenic complex. Nitrocellulose strips containing the separated antigens were incubated for 30 min with the samples diluted 1:51 in dilution buffer. Specific antibodies present in the sample bind to the RV antigens on the nitrocellulose strip. Bound antibodies were detected using an enzyme-labelled anti-IgG conjugate followed by substrate addition.

An IB result was classified as positive if the E1 band was present, regardless of E2 band visibility. An immunoblot was classified as negative if no bands were observed. If the IB result was negative, the sample was assigned a negative anti-RV IgG status. If the IB result was positive, the sample was assigned a positive anti-RV IgG status.

Although no universally accepted gold standard exists for rubella IgG detection, the immunoblot is widely used as a reference method because it enables the separate detection of antibodies against individual viral proteins and shows higher analytical sensitivity than commercial immunoassays, particularly in low-titer samples.

### 2.4. Data Analysis

For the calculation of sensitivity and specificity of each assay, the IB test was considered as the reference assay. Sensitivity was defined as the proportion of samples with a positive result in the cohort of IB-positive samples; specificity as the proportion of samples with a negative result in the group of IB-negative samples. The percentage agreement with the assigned status and 95% confidence intervals (95% CIs) were determined for each CIA. Because equivocal results occurred near the cut-off, performance was reported under two scenarios (equivocal interpreted as negative or as positive).

The quantitative test results reported by each CIA for samples with positive status were analyzed to compare differences in specific IgG among the five evaluated assays. The results were analyzed using repeated-measures statistical methods. Differences in median antibody levels were evaluated using the non-parametric Friedman test. Post hoc pairwise comparisons between assays were performed using the Wilcoxon signed-rank test. To account for multiple comparisons, *p*-values were adjusted using the Bonferroni correction. A *p*-value < 0.05 was considered statistically significant. Statistical analyses were performed using GraphPad Prism (version 10.0, GraphPad Software, San Diego, CA, USA).

## 3. Results

### 3.1. Serological Status with the Reference Assay (IB)

Since information on the vaccination status of the individuals from whom samples were obtained was unavailable, true positive and negative samples were classified according to the IB result. Of the 137 sera studied, 97 (70.8%) were classified as positive, 39 (28.4%) as negative, and 1 (0.7%) as equivocal. The serum sample found to be equivocal by IB was removed from the study. All IB-positive sera showed at least two bands: E1 and E1–E2.

### 3.2. Performance of CIAs

Qualitative results observed with the CIAs for all samples are summarized in Table 1. No assay yielded positive results in samples with a negative IB result. Only the Virclia and Euroimmun assays produced two and one equivocal results, respectively.

Of the 39 samples classified as seronegative, the Atellica, Alinity, and Euroimmun assays yielded negative results for all of them (100% specificity). The Virclia assay classified 37 sera as negative (94.9% specificity), and the Liaison assay classified 38 (97.4% specificity).

Of the 97 samples that tested positive by IB, 71 (73.2%) were positive in at least one of the evaluated assays, while only 18 (18.6%) showed positive results in all five assays. Using manufacturer-defined cut-offs, sensitivities in IB-positive samples were: Atellica 70.1% (68/97), Alinity 19.6% (19/97), Liaison 43.3% (42/97), Virclia 51.5% (50/97), and Euroimmun 46.4% (45/97). Notably, a substantial proportion of IB-positive samples fell within the equivocal zones of the assays (Table 1), indicating that discordance was mainly driven by grey-zone classification rather than by clear negative results. When equivocal results were interpreted as positive, sensitivities increased to 59.8–94.8% across platforms.

To improve readability, the sensitivity and specificity of each assay against the reference IB are summarized in Table 2 in two interpretative scenarios for equivocal results (equivocal interpreted as negative and equivocal interpreted as positive).

### 3.3. Concordance Between IB and CIA Qualitative Results

The agreement between the serological status assessed by IB and the qualitative outcomes (positive, equivocal, or negative) of the CIAs varied significantly, largely influenced by how equivocal results were interpreted. As shown in Table 3, when equivocal results were considered negative, agreement with IB ranged from 43% (Alinity) to 77% (Atellica). Reclassifying equivocal results as positive improved agreement to 71% and 96%, respectively.

### 3.4. Comparison of Quantitative Results of Immunoassays

**Figure 1:**
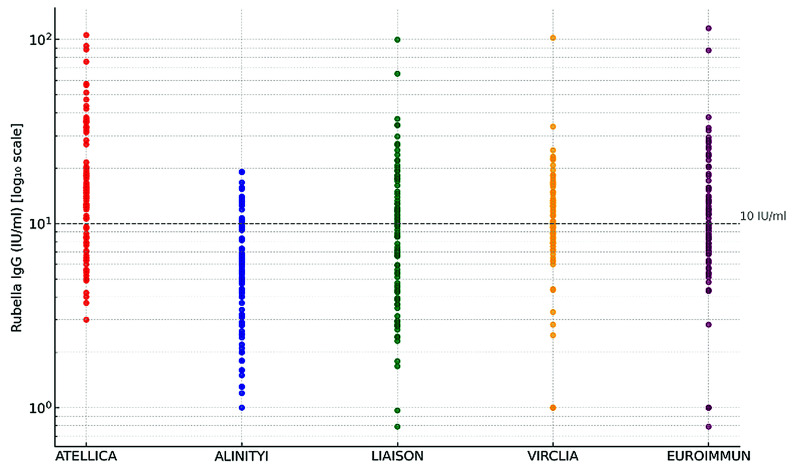
Dot histograms of the quantitative test result, expressed in log international units per millilitre (IU/mL), obtained from each of five commercial immunoassays, for a total of 97 immunoblot-positive samples. The assay’s cut-off is represented with a horizontal line.

All evaluated assays report results in IU/mL. Among samples testing positive by IB, rubella-specific IgG titers ranged from 1.0 to 19.1 IU/mL for Alinity, 3 to 105 IU/mL for Atellica, 0.8 to 99 IU/mL for Liaison, 1.0 to 101 IU/mL for Euroimmun, and 1.0 to 115 IU/mL for Virclia (Figure 1).

The statistical analysis using the Friedman test revealed significant differences in anti-rubella IgG levels measured by the five evaluated assays (*p* < 0.0001). Post hoc pairwise comparisons, performed using the Wilcoxon signed-rank test with Bonferroni correction, confirmed significant discrepancies between multiple assay pairs. Specifically, the Atellica assay yielded significantly higher IgG concentrations compared to all other methods (*p* < 0.001). In contrast, the Alinity assay consistently produced the lowest values.

## 4. Discussion

The determination of RV-specific IgG remains essential for epidemiological surveillance and for the prevention of congenital rubella. However, the lack of harmonization among commercial immunoassays continues to generate significant discrepancies, particularly in samples with low antibody titers. Despite the near elimination of rubella in many regions, assessing IgG levels remains critical for monitoring immunity and preventing congenital infections. In this study, we evaluated the performance of five CIAs for the detection of RV IgG, using IB as the reference method. A panel of 137 sera with negative or low-positive RV IgG levels was analyzed. Because the panel was intentionally enriched for low-titer samples, the sensitivity estimates reported here should be interpreted as reflecting performance in the diagnostic ‘grey zone’ rather than in an unselected population. Similar study designs focusing on low-reactivity sera have been used to specifically address inter-assay variability around the cut-off [12].

This panel was selected because discrepancies in this range are particularly relevant from both clinical and epidemiological perspectives. Moreover, its selection is justified by current epidemiological trends: the occurrence of sera with low IgG concentrations is increasingly common in countries with high vaccination coverage [14,15]. In such populations, low antibody concentrations may result from a weaker immune response elicited by vaccination compared with infection by the wild-type strain, or from the loss of the natural “booster” effect due to reduced viral circulation [16].

The IB used as the reference method shows high sensitivity. Antibodies against the E1 protein of RV are consistently detected in individuals after either natural infection or vaccination [17]. Furthermore, booster effects observed after revaccination of seronegative pregnant women—but with positive E1 responses on IB—support the high specificity of this assay [9].

All evaluated methods were calibrated against the WHO international standard RUBI-1-94. Our results confirm the findings reported by other authors regarding the low sensitivity and discrepancies among assays when testing samples with RV IgG concentrations close to the standard cut-off [12,13,18].

Using manufacturer-defined cut-offs and interpreting equivocal results as negative, specificity remained high (94.9–100%) while sensitivity was low (19.6–70.1%) in this low-titer panel, indicating that many immunoblot-positive sera would be reported as equivocal or negative by routine platforms. Thus, a large proportion of IB-positive samples with low RV IgG concentrations would have been classified as equivocal or negative (“non-immune status”), which could potentially lead to inappropriate clinical management. When equivocal results were interpreted as positive (i.e., removal of the grey zone), sensitivity increased markedly (59.8–94.8%), with minimal impact on specificity: Atellica, Alinity, and Euroimmun remained at 100%, whereas Liaison decreased to 97.4% and Virclia to 94.9%, consistent with a small number of immunoblot-negative specimens classified as equivocal by these assays (Table 2).

A pattern similar to that observed in the assays evaluated in our study has been reported for many other tests, including CLIA-based systems such as Architect (Abbott Diagnostics), Cobas 6000 (Roche Diagnostics), Vidas (bioMérieux), DxI (Beckman Coulter), Centaur (Siemens Healthcare), as well as ELISA-based assays such as Enzygnost (Siemens Healthcare), Vircell EIA (Vircell), and DiaSorin EIA (DiaSorin) [12,13].

The agreement between the CIAs tested and the status assigned by IB was low (ranging from 43% to 77%). This reflects the limitations of quantitative assays in detecting low antibody levels that remain detectable by more sensitive confirmatory methods such as IB. In our panel of samples, selected for having RV IgG values close to the cut-off, removing the equivocal zone and considering those results as positive markedly improved concordance (ranging from 71% for Alinity to 96% for Atellica) (Table 3).

Our results revealed marked quantitative differences among platforms, particularly in sera with values close to 10 IU/mL. Recent data from the College of American Pathologists revealed differences of up to one order of magnitude between manufacturers for the same specimen [19]. Siemens and Roche platforms tend to report the highest values, whereas Abbott and DiaSorin usually yield lower results [19]. This pattern was partially reproduced in our study: the Siemens platform provided the highest values, while Abbott yielded the lowest, and the Alinity assay tended to underestimate concentrations, placing more samples in the equivocal or negative range.

The discrepancies between CIAs may be attributed to the diversity of antigens, platforms, calibrations, and detection systems used. Some assays employ recombinant antigens (E1, E2), whereas others use whole-virus or viral lysate preparations [11,12,13]. In addition, the RUBI-1-94 standard [20] was developed from sera obtained after natural infection, not vaccination, which may affect its traceability in current populations [11,21]. This situation limits cross-platform comparability and questions the universal validity of the 10 IU/mL cut-off value.

Some authors recommend eliminating the equivocal zone and considering those results as positive, and performing, in cases with negative RV IgG results, a highly sensitive and specific assay such as the IB [22]. In a recent study conducted in Canada, it was estimated that in populations where the majority have been vaccinated, antibody levels below 2–5 IU/mL are more indicative of a true lack of immunity, suggesting that the conventional cut-off of 10 IU/mL may be too high for these populations [14]. In fact, many countries, including Finland (<4 IU/mL seronegative, 4–7 IU/mL equivocal) and Australia (<4 IU/mL seronegative, 4–15 IU/mL equivocal), already use lower cut-off values for population screening [15,23].

A limitation of this study is that individual vaccination status and time since vaccination were not available. Consequently, we could not stratify results by vaccination history or distinguish vaccine-induced from infection-induced antibody profiles, which may influence low-titer distributions and the frequency of equivocal classifications across assays.

This lack of reproducibility across assays may have important clinical and epidemiological implications. In pregnant women, incorrect classification of immune status can lead to unnecessary follow-up or revaccination. The possibility of assigning different classifications (positive, equivocal, or negative) to the same sample when tested on different platforms highlights the need to perform all tests for a given individual—especially in pregnant women—using the same assay. At the population level, it may result in underestimation of immunity and inappropriately designed vaccination strategies. Such heterogeneity could compromise both clinical decision-making and seroepidemiological assessments.

Taken together, these findings support re-evaluating current manufacturer cut-offs in the context of post-vaccination antibody profiles and reinforce the need for improved harmonization across platforms, including calibration alignment and clearer interpretive criteria for borderline results. As recently emphasized, decades of experience indicate that standardization of quantitative rubella IgG results has remained ineffective, underscoring the need for assays that reliably distinguish true absence from low-level reactivity [24]. Until such harmonization is achieved, laboratories should explicitly report the interpretive criteria applied, and clinicians and public health programs should interpret low or borderline rubella IgG concentrations cautiously, considering confirmatory testing and/or follow-up strategies when results may affect clinical or epidemiological decision-making.

## 5. Conclusions

In low-titer rubella IgG samples near the cut-off, commercially available immunoassays show substantial variability—largely due to grey-zone classifications—so borderline results are not interchangeable across platforms and should be interpreted cautiously, ideally with confirmatory testing or follow-up when clinically relevant.

## Figures and Tables

**Table 1:** Qualitative test results obtained from five commercial immunoassays compared with an anti-rubella virus IgG status.

	No. (%) of Samples with Indicated Assay Result in 39 IB Negative Samples			No. (%) of Samples with Indicated Assay Result in 97 IB Positive Samples		
Assay	Positive	Negative	Equivocal	Positive	Negative	Equivocal
Atellica	0	39 (100)	0	68 (70.1)	5 (5.2)	24 (24.7)
Alinity	0	39 (100)	0	19 (19.6)	39 (40.2)	39 (40.2)
Liaison	0	38 (97.4)	1 (2.6)	42 (43.3)	28 (28.9)	27 (27.8)
Virclia	0	37 (94.9)	2 (5.1)	50 (51.5)	10 (10.3)	37 (38.1)
Euroimmun	0	39 (100)	0	45 (46.4)	32 (33)	20 (20.6)

IB: immunoblot.

**Table 2:** Sensitivity and specificity of evaluated rubella IgG assays versus Immunoblot (IB) under two equivocal-handling scenarios (with 95% CI).

Assay	Sensitivity (%, 95% CI) (Equivocal → Negative)	Specificity (%, 95% CI) (Equivocal → Negative)	Sensitivity (%, 95% CI) (Equivocal → Positive)	Specificity (%, 95% CI) (Equivocal → Positive)
Atellica	68/97 (70.1%; 60–79)	39/39 (100.0%; 91.0–100.0)	92/97 (94.8%; 88.4–98.3)	39/39 (100.0%; 91.0–100.0)
Alinity	19/97 (19.6%; 12.2–28.9)	39/39 (100.0%; 91.0–100.0)	58/97 (59.8%; 49.3–69.6)	39/39 (100.0%; 91.0–100.0)
Liaison	42/97 (43.3%; 33.3–53.7)	39/39 (100.0%; 91.0–100.0)	69/97 (71.1%; 61.0–79.9)	38/39 (97.4%; 86.5–99.9)
Euroimmun ELISA	45/97 (46.4%; 36.2–56.8)	39/39 (100.0%; 91.0–100.0)	65/97 (67.0%; 56.7–76.2)	39/39 (100.0%; 91.0–100.0)
Virclia	50/97 (51.5%; 41.2–61.8)	39/39 (100.0%; 91.0–100.0)	87/97 (89.7%; 81.9–94.9)	37/39 (94.9%; 82.7–99.4)

Sensitivity was calculated among IB-positive samples (n = 97) and specificity among IB-negative samples (n = 39). 95% CI: 95% confidence intervals. Equivocal results were interpreted as negative (scenario 1) or as positive (scenario 2), as indicated.

**Table 3:** Concordance between IB and CIA results from the 136 serum samples according to different interpretation of equivocal results.

	% (CI 95%) of Concordant Sera by Indicated CIA				
Interpretation of CIA Equivocal Results	Atellica	Alinity	Liaison	Virclia	Euroimmun
Equivocal interpreted as negative	77 (69–83)	43 (35–51)	59 (50–67)	65 (57–73)	62 (53–70)
Equivocal interpreted as positive	96 (92–98)	71 (63–78)	79 (71–85)	91 (85–95)	76 (69–83)

CI: confidence intervals; CIA: commercial immunoassays.

## Data Availability

The raw data supporting the conclusions of this article will be made available by the authors on request.

## Supplementary Materials

The following supporting information can be downloaded at: https://www.mdpi.com/article/10.3390/microorganisms14010058/s1, Table S1: Characteristics of commercial immunoassays included in the study CIA.
