# Supplementary material for: Evaluation of Commercial Immunoassays for Rubella Virus IgG Detection in Low-Antibody Sera Using a Recombinant Immunoblot as a Reference Method
Source: Microorganisms. 2025 Dec 26;14(1):58. doi: 10.3390/microorganisms14010058 (PMC12844522; doi:10.3390/microorganisms14010058)
Supplement: Supplementary file 1 [file microorganisms-14-00058-s001.zip › microorganisms-4027988-supplementary.pdf]

Table S1. Characteristics of commercial immunoassays included in the study CIA

Assay	Solid-phase type	Antigen	Detection system	Standard	Range and interpretation of results (IU/mL)
Alinity i Rubella IgG (Abbott Diagnostics)	Paramagnetic microparticles coated with partially purified rubella virus	Partially purified rubella virus	Chemiluminescent microparticle immunoassay (CMIA); acridinium-labeled anti-human IgG	Traceable to WHO 1st International Standard for anti-rubella immunoglobulin (NIBSC RUBI-1-94)	0.0–4.9: negative; 5.0–9.9: equivocal (grey zone); ≥10.0: positive
Atellica IM Rub G (Siemens Healthineers)	Paramagnetic particles coated with mouse monoclonal anti-human IgG Fc antibodies	Inactivated rubella virus antigen (strain HPV77), acridinium-ester labeled	Chemiluminescence (acridinium ester)	Traceable to WHO 1st International Standard for anti-rubella immunoglobulin (NIBSC RUBI-1-94)	<5.0: nonreactive; 5.0–<10.0: equivocal; ≥10.0: reactive
LIAISON® Rubella IgG II (DiaSorin)	Magnetic particles coated with inactivated rubella viral particles	Inactivated rubella viral particles (strain HPV77)	Chemiluminescence; anti-human IgG monoclonal antibody and isoluminol sustrate	Calibrators traceable to WHO 1st International Standard for anti-rubella immunoglobulin (NIBSC RUBI-1-94)	<5.0: negative; 5.0–<10.0: equivocal; ≥10.0: positive
Anti-Rubella Virus ELISA (IgG) (EUROIMMUN)	Antigen-coated microplate wells	Purified rubella virus antigens from inactivated Vero cell lysates infected with HPV-77 strain	ELISA: peroxidase-labeled anti-human IgG; TMB substrate	Calibration in IU using NIBSC RUBI-1-94 (Anti-Rubella Serum, 1st International Standard)	<8: negative; ≥8–<11: borderline; ≥11: positive
VirClia® Rubella IgG (Vircell)	Antigen-coated polystyrene wells	Inactivated rubella antigens	Chemiluminescence; anti-human IgG monoclonal antibody and isoluminol sustrate	WHO 1st International Standard for anti-rubella immunoglobulin	<5.0: nonreactive; 5.0–<10.0: equivocal; ≥10.0: reactive
